# Overlapping spatial clusters of sugar-sweetened beverage intake and body mass index in Geneva state, Switzerland

**DOI:** 10.1038/s41387-019-0102-0

**Published:** 2019-11-14

**Authors:** Stéphane Joost, David De Ridder, Pedro Marques-Vidal, Beatrice Bacchilega, Jean-Marc Theler, Jean-Michel Gaspoz, Idris Guessous

**Affiliations:** 1 Laboratory of Geographic Information Systems (LASIG), School of Architecture, Civil and Environmental Engineering (ENAC), École Polytechnique Fédérale de Lausanne (EPFL), Geneva, Switzerland; 2 Unit of Population Epidemiology, Division of Primary Care Medicine, Department of Primary Care Medicine, Geneva University Hospitals, Geneva, Switzerland; 3 Group of Geographic Information Research and Analysis in Population Health (GIRAPH), Geneva, Switzerland; 4 La Source, School of Nursing, University of Applied Sciences and Arts Western Switzerland (HES-SO), Lausanne, Switzerland; 5 Faculty of Medicine, University of Geneva, Geneva, Switzerland; 6 Department of Medicine, Internal Medicine, Lausanne University Hospital, Lausanne, Switzerland

**Keywords:** Obesity, Risk factors, Epidemiology

## Abstract

**Background:**

Obesity and obesity-related diseases represent a major public health concern. Recently, studies have substantiated the role of sugar-sweetened beverages (SSBs) consumption in the development of these diseases. The fine identification of populations and areas in need for public health intervention remains challenging. This study investigates the existence of spatial clustering of SSB intake frequency (SSB-IF) and body mass index (BMI), and their potential spatial overlap in a population of adults of the state of Geneva using a fine-scale geospatial approach.

**Methods:**

We used data on self-reported SSB-IF and measured BMI from residents aged between 20 and 74 years of the state of Geneva (Switzerland) that participated in the Bus Santé cross-sectional population-based study (*n* = 15,423). Getis-Ord Gi spatial indices were used to identify spatial clusters of SSB-IF and BMI in unadjusted models and models adjusted for individual covariates (education level, gender, age, nationality, and neighborhood-level median income).

**Results:**

We identified a significant spatial clustering of BMI and SSB-IF. 13.2% (*n* = 2034) of the participants were within clusters of higher SSB-IF and 10.7% (*n* = 1651) were within clusters of lower SSB-IF. We identified overlapping clusters of SSB-IF and BMI in specific areas where 11.1% (*n* *=* 1719*)* of the participants resided. After adjustment, the identified clusters persisted and were only slightly attenuated indicating that additional neighborhood-level determinants influence the spatial distribution of SSB-IF and BMI.

**Conclusions:**

Our fine-scale spatial approach allowed to identify specific populations and areas presenting higher SSB-IF and highlighted the existence of an overlap between populations and areas of higher SSB-IF associated with higher BMI. These findings could guide policymakers to develop locally tailored interventions such as targeted prevention campaigns and pave the way for precision public health delivery.

## Introduction

The prevalence of obesity and obesity-related diseases has been increasing steadily in most countries over the past decades^1^. Although pathways leading to obesity are varied and complex, notably because of the interplay of genetic, environmental, and social factors, it has been suggested that the consumption of sugar-sweetened beverages (SSBs) is an important contributory factor^2,3^. SSBs are defined as drinks with added sugar and include a wide range of products such as soft drinks, flavored juice drinks, sports drinks, sweetened tea, coffee drinks, energy drinks, and electrolyte replacement drinks^4^. While these beverages present significant differences in sugar content, drinks such as sodas can contain up to 39 g of sugar per 330 ml can^5^. Worldwide, SSB sales and consumption have increased these last decades with the greatest consumptions reported in Argentina and the USA^6^. In Europe, the annual per capita consumption of SSB was about 95 l in 2015^7^. In Switzerland, SSB consumption increased during the last 20 years among children, teenagers, and adults to reach an annual consumption of about 80 l^8^ thus representing a growing contribution to the total caloric intake. Although some controversy remains on whether the association between obesity and SSB consumption is causal, a recent systematic review and meta-analysis of large prospective cohort studies and randomized controlled trials concluded that SSB consumption promotes weight gain in children and adults^9^. In addition to the increase in energy intake associated with long term weight gain, SSBs may also cause health risks through the metabolic response to fructose, a major component of SSBs. High intake of fructose can lead to increased visceral adiposity, lipid dysregulation, and decreased insulin sensitivity^10^. Recent meta-analyses also concluded that a normal consumption of SSBs was associated with a greater risk of developing diet-related diseases such as cardiovascular diseases^11^, hypertension^12,13^, stroke^14,15^, and type 2 diabetes^16^, independently of adiposity.

Accordingly, several governmental and public health interventions have been implemented to reduce the consumption of SSB and increase awareness about the health consequences associated with SSB consumption^17^. In the last decade, SSB taxation, already introduced in some states in the USA and several other countries, has been proposed to reduce SSB consumption, reduce healthcare costs and generate revenue for health initiatives^18^. However, objections against such tax have raised in many countries, notably due to its regressive nature and supposed lack of efficacy to lower obesity prevalence^18^. Nevertheless, it has been suggested that these arguments could be addressed by ensuring that the revenues generated are allocated preferentially to programs promoting nutrition and obesity-prevention for the most in need^18^. Therefore, identifying specific populations at risk of SSB overconsumption is of utmost importance for health policymakers. Still, the identification of such populations or areas in need of intervention is far from optimal.

Spatial analysis methods have been developed and introduced in epidemiological research to explore the link between place of residence and health^19^. Areas where individuals show a higher BMI and a high need for interventions to reduce SSB consumption can be revealed by spatial clustering, defined as an unusual concentration of individuals with a specific outcome in space. Although the identification of these populations and areas could guide place-based public health interventions, research on the spatial variation of diet-related diseases risk factors such as SSB consumption at the local level remains scarce. Most studies focused on SSB consumption patterns at the county or state-level by aggregating individual-level data^20–22^ which results in a smoothing altering the original signal^23^. One recent study reported the spatial clustering of SSB consumption in adolescents using a sample of 1292 precisely georeferenced residential addresses from the Boston youth study and identified clustering of high prevalence of non-soda SSBs intake^24^. However, this study examined SSB consumption as a binary variable (never versus any) and did not examine the spatial clustering of BMI.

The primary objective of this study was to investigate whether local spatial clusters of SSB-IF and BMI exist among a general adult population of the state of Geneva. The secondary objective was to investigate if clusters of higher SSB-IF overlap with clusters of higher BMI. Areas of high BMI and high SSB consumption—i.e., areas of high priority for future place-based interventions to reduce SSB consumption could be selected by policymakers as the starting point in developing locally tailored interventions.

## Methods

### Data source and study population

Data on adults were collected using the Bus Santé study^25^, a cross-sectional population-based study that collects information on cardiovascular risk factors. Every year, a stratified sample of 500 men and 500 women—representative of the State of Geneva’s 100,000 males and 100,000 females non-institutionalized residents aged 35–74 (20–74 since 2011)—is recruited and studied. Four trained collaborators interview and examine the participants. All procedures are reviewed and standardized across technicians regularly.

Eligible subjects are identified via a standardized procedure using an annual residential list established by the local government. This list includes all individuals living in the State of Geneva. An invitation to participate is mailed to the sampled subjects and, if they do not respond, up to seven telephone calls are made at different times on various days of the week. If telephone contact is unsuccessful, two more invitation letters are sent. Subjects that are not reached are replaced using the same selection protocol, the ones who refuse to participate are not replace. Finally, subjects who accept to participate receive a self-administered standardized questionnaire, including a semi-quantitative food frequency section. Geographic coordinates of the postal address are used for individual geographic information. For this analysis, data from surveys 1995 to 2014 were used, corresponding to 15,767 participants. The average participation rate for 1995–2014 was 61% (range: 53–69%).

### Body mass index and sugar-sweetened beverages intake frequency

Participants bring filled-in questionnaires, which are checked for correct completion by trained interviewers^26^. Body weight is measured with the subject lightly dressed, without shoes and using a medical scale (precision 0.5 kg); standing height is measured using a medical gauge (precision 1 cm). Body mass index (BMI) is calculated as weight (kg)/height (m^2^).

SSB-IF is assessed for every participant using a self-administered, semiquantitative food frequency questionnaire (FFQ), which also included portion size^27,28^. This FFQ has been validated against 24-h recalls among 626 volunteers from the Geneva population, and data derived from this FFQ have recently contributed to worldwide analyses^29,30^. Briefly, this FFQ assesses the dietary intake of the previous four weeks and consists of 97 different food and beverage items, including SSB (colas, sodas, lemonades, syrups). For each item, consumption frequencies ranging from “less than once during the last four weeks” to “2 or more times per day” were provided; daily SSB-IF was computed from 0 for “less than once during the last 4 weeks” to 2.5 for “2 or more times per day”. The local Institutional Ethics Committee approved the study. All participants gave written informed consent before entering the study.

### Covariates

SSB consumption and BMI covariates included education level, gender, age, nationality, and the neighborhood-level median income of the area. The Bus Santé study data was used to assess education level, gender, age, and nationality. Education level was dichotomized as having tertiary education or not; gender was defined as either male or female; age was defined as a continuous variable; nationality was dichotomized as having Swiss nationality or not. We used income data characterizing the 475 Geneva statistical sectors in 2009 for adjustment. These data were produced by *Statistique Genève*^31^. The yearly income value (1 CHF = 1.007 USD, June 2018) was attributed to Bus santé participants based on their postal address within the corresponding statistical sector.

### Statistical analyses

A median regression analysis was used to obtain the SSB-IF and the BMI adjusted for education level, gender, age, nationality, and the median income of the area^32^.

Using the geographical coordinates of the place of residence, we used the Getis-Ord Gi statistic^33^ to investigate whether SSB-IF and BMI were spatially dependent. Getis-Ord Gi indicators are statistics that measure spatial dependence and evaluate the existence of local clusters—hot or cold spots—in the spatial arrangement of a given variable, here SSB-IF and BMI. Gi indicators compare the local sum of individual SSB-IF values included within a given spatial buffer proportionally to the sum of individual SSB-IF values within the whole study area, and similarly for BMI^34^. The Gi statistic returned for each value is a *Z*-score to which a p-value is associated. The null hypothesis for this statistic is that the values being analyzed exhibit no spatial clustering. When the *p*-values are statistically significant, it can be assumed that the spatial distribution is not random. Statistical significance testing was based on a conditional randomization procedure^35^ using a sample of 999 permutations. Large statistically significant positive *Z*-scores reveal a clustering of higher values while large significant negative Z-scores reveal a clustering of lower values.

We assessed the presence of overall spatial dependence using the global Moran’s I statistic^36^. A correlogram calculated with a maximum distance of 4 km produced global Moran’s I ranging between 0 and 0.011 for BMI in the ten 400 m-bins, and between 0 and 0.001 for SSB-IF. Considering a correlogram calculated with a maximum distance of 2 km, Moran’s I for BMI ranged between 0.002 and 0.016 in the ten 200 m-bins, and between 0 and 0.003 for SSB-IF, translating no global spatial autocorrelation in both variables. Therefore, no distance threshold could be determined on this basis. The results of the analysis of SSB-IF and BMI variables presented in this study used a binary spatial weights matrix based on a fixed spatial buffer of 1200 m around the place of residence of each individual as this distance approximates the size of a typical neighborhood in the urban areas of the studied territory. The spatial weights were row standardized—the sum of the weights (W) equals 1, and each individual weight equals 1/W_i_—to obtain proportional weights. This method is used when the number of neighbors varies. In our analysis, the indicators Gi and Gi* are homogeneous of order zero in W_ij_ and thus invariant^34^. Statistical significance was considered for a *p*-value < 0.05 for all spatial dependence measures.

Finally, to determine whether SSB-IF, BMI, and their spatial dependence were stable during the 1995–2014 period, the dataset was divided into 3 subperiods with a high number of participants to favor the robustness of the evaluation: subperiod 1 (P1) = 1995–2001 (*n* = 5511), subperiod 2 (P2) = 2002–2008 (*n* = 4714), and subperiod 3 (P3) = 2009–2014 (*n* = 5357). We then conducted a Tukey’s multiple comparison analysis^37^ method to ensure that the mean of the SSB-IF and the BMI had not increased or decreased sharply between the three subperiods. Finally, we calculated global Moran’s I^36^ statistics to verify that there was no difference in global spatial autocorrelation between the three subperiods.

On the maps produced, white dots correspond to individuals with a non-significant Z-score. Individuals with a statistically significant positive *Z*-score are represented by red dots, indicating a clustering of higher values within a spatial buffer of 1200 m, and are found closer together than expected if the underlying spatial process was random. Blue dots correspond to individuals with a statistically significant negative *Z*-score, meaning that lower values cluster within a spatial buffer of 1200 m, and are found closer together than expected if the underlying spatial process was random. We present maps of SSB-IF and BMI in unadjusted models and models adjusted for individual covariates.

In order to compare the overlap between SSB-IF and BMI spatial clusters, participants were categorized in 10 classes (Fig. S3) using the combination of the previously computed Getis-Ord Gi clusters SSB-IF and BMI. The same classification was performed before and after adjustment for covariates.

## Results

After excluding participants for missing data, 15,423 (97.8%) participants were retained. Genders were represented at the same rate (50.0%), the mean age of the participants was 51.3 years (SD ± 11.0 years), 37.7% of the participants had a university level degree, 70.6% were of Swiss nationality and 29.4% of other nationalities, the neighborhood-level median yearly income was 72,166 CHF. The mean SSB-IF was about 0.22 SSB/day (SD ± 0.5 SSB/day) and 0.18 SSB/day (SD ± 0.5 SSB/day) after adjustment for covariates (Table 1). About 49% of the participants reported any consumption of SSB in the past four weeks. SSB consumption prevalence was around 70% in participants under 40 years old. The prevalence of participants consuming SSBs “once a day” and “twice or more per day” was 5.8% and 3.5%, respectively. The mean BMI was 24.9 kg/m^2^ (SD ± 4.1 kg/m^2^) and 25.0 kg/m^2^ (SD ± 3.8 kg/m^2^) after adjustment for covariates. Around half of participants had a normal weight (49.5%, BMI between 18.5 and 25 kg/m^2^) while 44% were at least overweight.

**Table 1:** Summary characteristics, 1995–2014 Bus Santé study participants (*n* = 15,423)

Variable	*n* (%)	Mean (SD)
Gender		
Men	7713 (50)	–
Women	7710 (50)	–
Age (years)	15,423 (100)	52.3 (11.0)
Neighborhood-level median income (CHF)	15,423 (100)	765,43.8 (19961.4)
Education		
Tertiary	5820 (37.8)	–
Others	9603 (62.2)	–
Nationality		
Swiss	10,883 (70.5)	–
Others	4540 (29.5)	–
Body mass index (kg/m^2^)	15,423 (100)	24.9 (4.0)
Sugar-sweetened beverage intake (SSB per day)	15,423 (100)	0.2 (0.5)

Analyses of mean and trends of BMI and SSB-IF (Table S1), as well as Moran’s I (Table S2) across the three subperiods are presented in the supplementary materials. Despite a slight increase of BMI and SSB-IF over time, the difference was only significant between P1 (1995–2001) and P3 (2009–2014) for both raw and adjusted variables (Fig. S1). The absence of global spatial autocorrelation for both variables was stable during the three subperiods while the spatial distribution of local clusters of BMI and SSB-IF slightly varied (SSB-IF hotspot downtown during P1 only) (Fig. S2).

### Sugar-sweetened beverages intake clusters

**Fig. 1:**
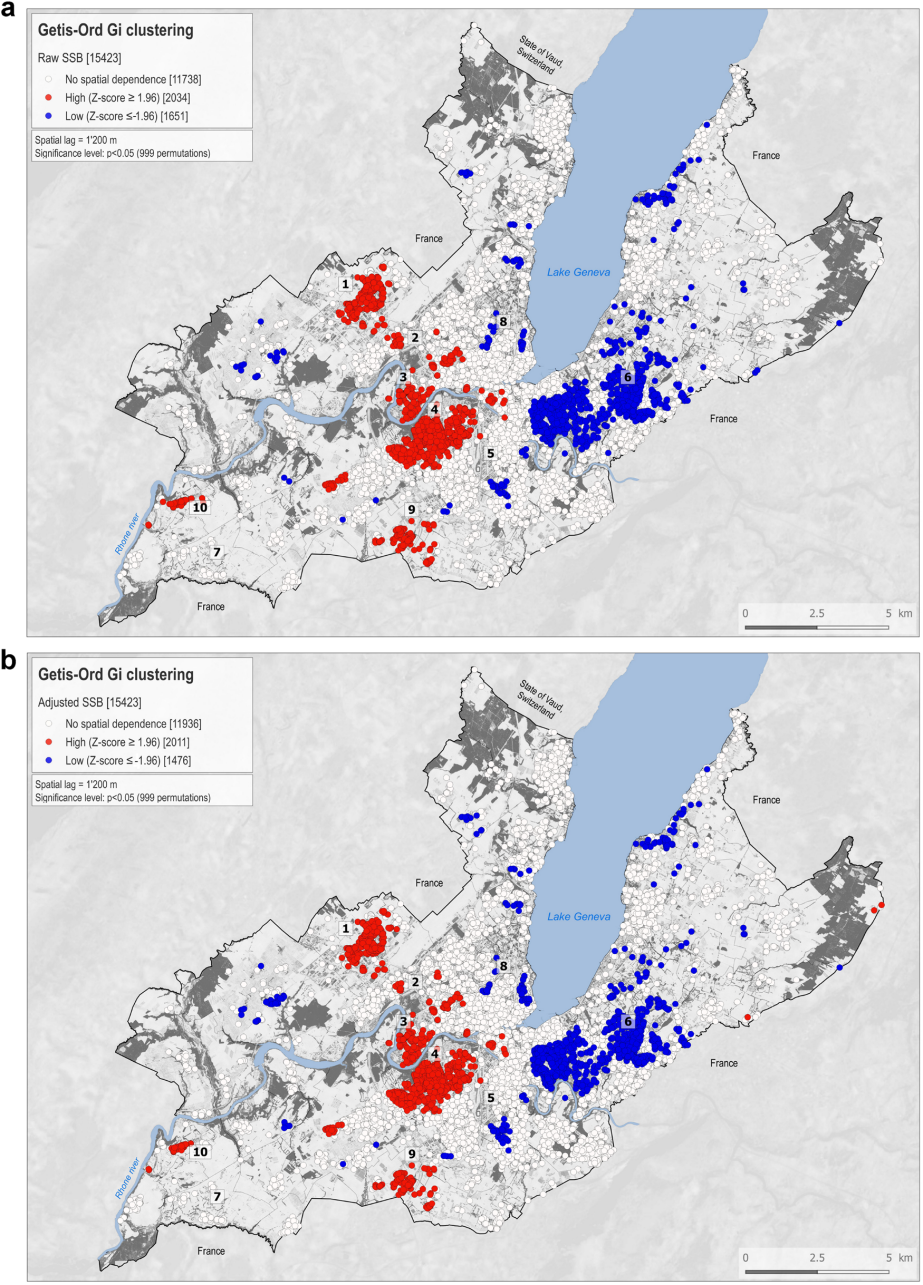
Spatial clustering of SSB-IF. Getis-Ord Gi clusters calculated for 15,423 Bus santé participants (1995–2014) for the raw sugar-sweetened beverage (SSB) intake variable (**a**) and adjusted for covariates (**b**). White dots correspond to individuals with a non-significant *Z*-score. Red dots correspond to individuals with a statistically significant positive *Z*-score (*α* = 0.05), meaning that higher values cluster within a spatial buffer of 1200 m and are found closer together than expected if the underlying spatial process was random. Blue dots correspond to individuals with a statistically significant negative *Z*-score (*α* = −0.05), meaning that lower values cluster within a spatial buffer of 1200 m and are found closer together than expected if the underlying spatial process was random. Indicative landmarks numbered 1–10 are displayed on the maps and used to support the description of the results

Before adjustment, 13.2% of the participants (*n* = 2034) were part of a cluster of higher SSB-IF, 10.7% (*n* = 1651) of a cluster of lower SSB-IF (Fig. 1a) and 76.1% (*n* = 11,738) showed no spatial dependence. After adjustment, 13.0% (*n* = 2011) of the participants were included within SSB-IF hot spots, 9.6% (*n* *=* 1476) in SSB-IF cold spots (Figs. 1b) and 77.4% (*n* = 11,936) showed no spatial dependence (Fig. S4A). Both analyses highlighted clear spatial patterns of SSB-IF with SSB-IF cold spots mainly located to the east of the lake (Fig. 1, landmark #6) and SSB-IF hot spots to the west (Fig. 1, landmarks #1, #3, #4, #9). The main effect of covariates adjustment was an attenuation of the geographic footprint of hot and cold spots.

### BMI clusters

**Fig. 2:**
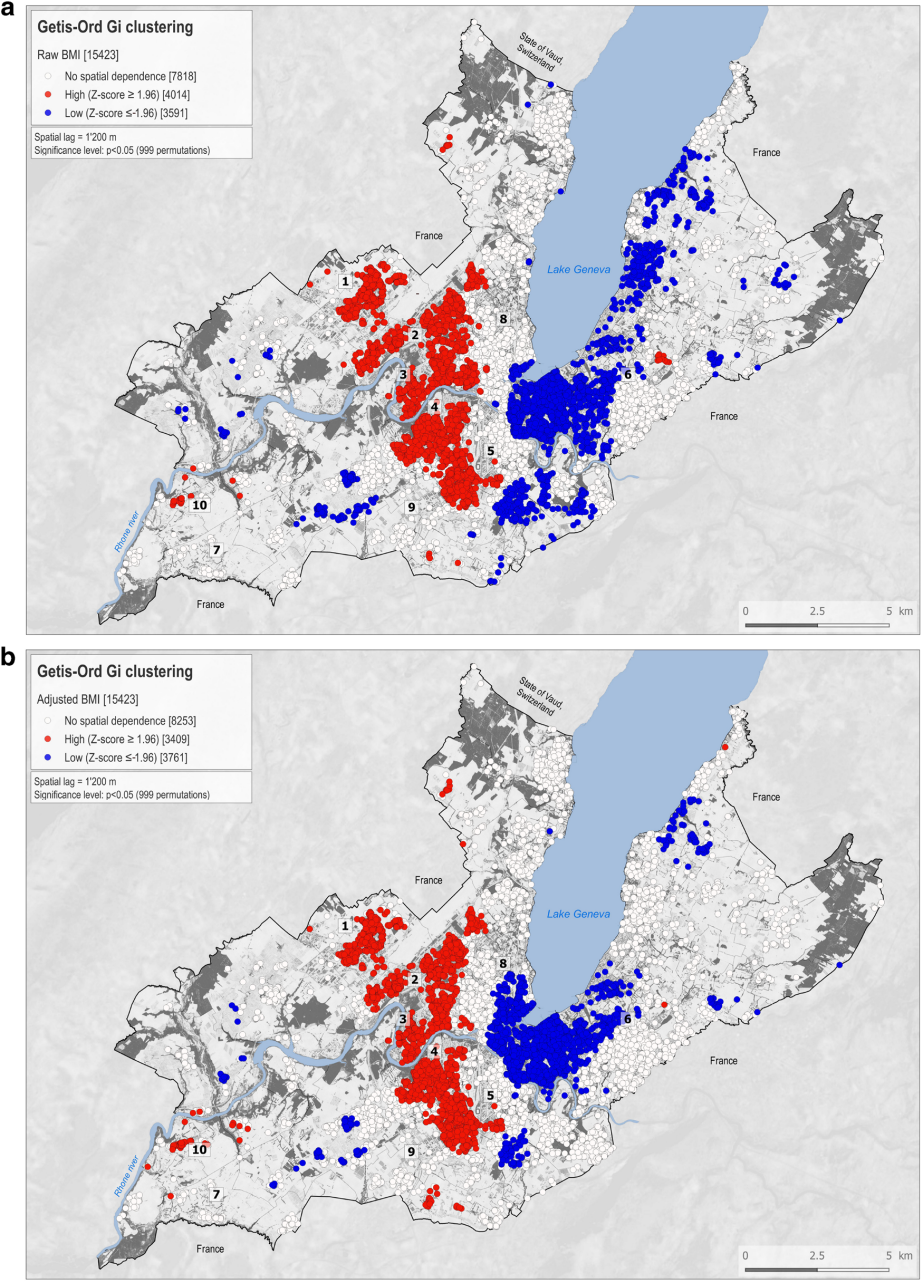
Spatial clustering of BMI. Getis-Ord Gi clusters calculated for 15,423 Bus santé participants (1995–2014) for the raw body mass index (BMI) variable (**a**) and adjusted for covariates (**b**). White dots correspond to individuals with a non-significant *Z*-score. Red dots correspond to individuals with a statistically significant positive *Z*-score (*α* = 0.05), meaning that higher values cluster within a spatial buffer of 1200 m and are found closer together than expected if the underlying spatial process was random. Blue dots correspond to individuals with a statistically significant negative *Z*-score (*α* = −0.05), meaning that lower values cluster within a spatial buffer of 1200 m and are found closer together than expected if the underlying spatial process was random. Indicative landmarks numbered 1–10 are displayed on the maps and used to support the description of the results

Before adjustment, 26.0% (*n* = 4014) of the participants were located within BMI hot spots, 23.3% (*n* = 3591) within BMI cold spots and 50.7% (*n* = 7818) showed no spatial dependence (Fig. 2a). After adjustment, 22.1% (*n* = 3409) were located within BMI hot spots, 24.4% (*n* = 3761) within BMI cold spots (Fig. 2b) and 53.5% (*n* = 8253) showed no spatial dependence (Fig. S4B). The adjustment of covariates thinned down the large hot spot located between landmarks #1 and #5 and shifted the large cold spot located between landmarks #5 and #6 towards the west, while reducing it slightly.

### Spatial overlap between SSB-IF and BMI clusters

**Fig. 3:**
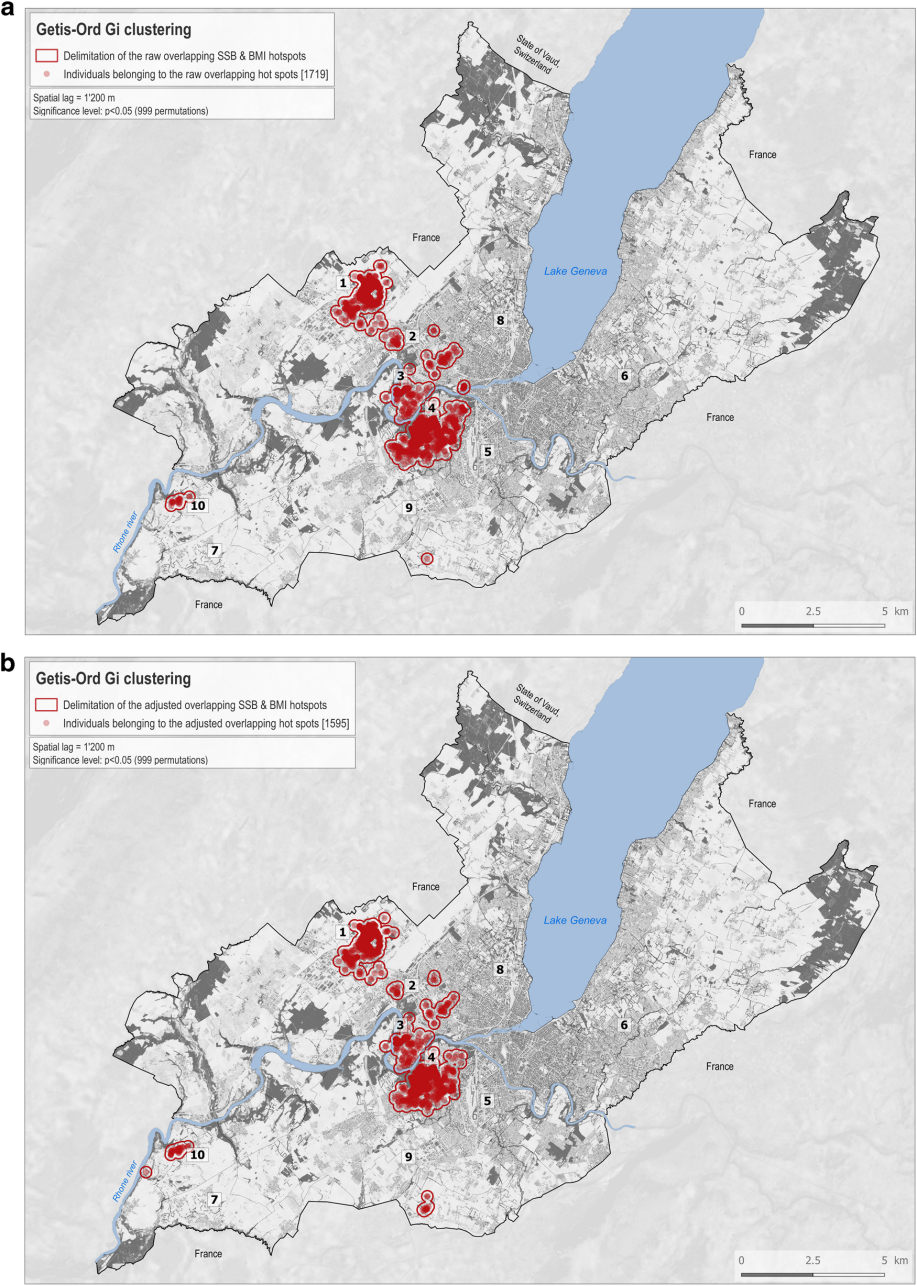
Overlap of higher SSB and higher BMI spatial clusters. The main delimited clusters with individuals belonging to both raw SSB-IF and raw BMI hotspots contain 1719 individuals. **a** The main delimited clusters with individuals belonging to the adjusted SSB-IF and BMI hotspots contain 1595 individuals. **b** Indicative landmarks numbered 1–10 are displayed on the maps and used to support the description of the results

Spatial overlap between high SSB-IF and high BMI clusters (i.e., a co-location of SSB-IF hot spot and BMI hot spot-class 1) of around 40% was identified and included 11.1% (*n* = 1719) of the participants (Fig. 3a). After adjustment for education level, gender, age, nationality, and the median income of the area, the overlap between high SSB-IF and high BMI clusters was around 42% and included 10.3% (*n* = 1595) of the participants (Fig. 3b). The overlap between lower SSB-IF and lower BMI clusters was around 25% in the unadjusted model and around 19% after adjustment for covariates. The overlap between discordant clusters—higher BMI with lower SSB-IF and lower BMI with higher SSB-IF—was very low (<1%).

## Discussion

This study reveals statistically significant spatial clusters of measured BMI and self-reported SSB-IF among a general adult population using novel spatial statistical methods. Spatial clusters of SSB-IF and BMI resisted the adjustment for covariates and were only slightly attenuated. We identified overlapping spatial clusters of SSB-IF and BMI in specific areas in both unadjusted models and models adjusted for covariates. The intersection between the set of participants in higher BMI clusters and the set of participants in higher SSB-IF clusters was around 40% and included around one-tenth of the participants. Interestingly, around 80% of the participants in the cluster of higher adjusted SSB-IF were also in the cluster of higher adjusted BMI while only 53% of the participants in the cluster of higher adjusted BMI were also in the cluster of higher adjusted SSB-IF.

To the best of our knowledge, this is the first study to examine SSB-IF and BMI spatial clustering simultaneously using a fine-scale geospatial approach. Other studies explored the spatial distribution of SSB consumption but at broader geographic scales, usually county or state-level^20–22^. However, the analysis of local-scale phenomena, such as SSB consumption, can be biased by aggregating point-based measures into large administrative spatial units^23^. By using individual data and considering space as a continuum, this study addresses the spatial aggregation bias.

Some of the identified areas presenting a higher BMI and SSB-IF have a lower socioeconomic status than other districts of Geneva, an urban state where recent evidence highlighted the existence of social inequalities in dietary intake^38^. Whilst neighborhood socioeconomic status (e.g., neighborhood deprivation, neighborhood segregation, population density) is known to be a determinant of dietary habits, obesity, obesity-related diseases and even mortality^39^, we also identified SSB-IF clusters after adjustment for education level, gender, age, nationality, and neighborhood-level median income suggesting that other factors such as network phenomena^40^ (e.g., social networks) and environmental factors^41^ (e.g., types of food stores, food access) influence weight status and SSB consumption. In 2017, Tamura et al. reported the spatial clustering of self-reported SSB consumption in adolescents using a sample of 1292 precisely georeferenced residential address from the Boston youth study^24^. They identified a single cluster of high prevalence of non-soda SSB consumption. Interestingly, the spatial cluster of non-soda SSB they detected did not resist adjustment for gender, education, age, and ethnicity, suggesting that the covariates played a larger role in the determination of the spatial distribution of the prevalence of SSB consumption.

In line with our previous work^42^, we found spatial clustering of BMI in adults from the general population and compared them to SSB intake frequency clusters. We found significant spatial overlap between higher SSB-IF and higher BMI clusters, and between lower SSB-IF and lower BMI. The overlap of about 40% identified between high SSB-IF and high BMI compared to only 1% between discordant clusters brings further evidence on the link between SSB consumption and weight status. However, this study being the first to examine simultaneously BMI and SSB consumption, we are lacking evidence to compare the extent of the spatial overlap. The areas of high BMI combined with high SSB-IF could be interpreted as areas where individuals are already suffering the negative impact of SSB intake frequency on weight and potentially other related diseases. Although these areas could also be explained by the co-location of environmental and social determinants of SSB consumption and weight status. Further research on the geographical patterns of their shared causal mechanisms is needed.

Our study is not without limitations. Firstly, regarding spatial statistic parameters, we defined our spatial weights matrix using a spatial buffer of 1200 m, but other choices may produce slightly different results. We tested the robustness of our findings using different spatial buffers and found no meaningful difference in the clusters obtained. Secondly, we favored the Getis-Ord Gi statistic over the Local Indicators of Spatial Association^35^ as we focused primarily on the detection of local clusters of higher and lower values, the study of discordant behaviors would be of interest for future investigations. Thirdly, participants and non-participants in the Bus Santé study may differ regarding SSB consumption, and participation bias cannot be excluded. Still, to reduce participation bias, the Bus Santé study has a mobile examination unit that covers three major areas of the State facilitating the participation of people living in disadvantaged areas. Fourthly, SSB-IF was self-reported and recall, as well as social desirability biases cannot be excluded. Finally, preliminary analysis of 3 temporal groups (1995–2001; 2002–2008; 2009–2014) of SSB-IF and BMI produced a similar overall spatial structure as translated by a stable global Moran’s I over time; local Getis-Ord Gi clusters are also stable, with the only exception of SSB-IF for P1 exhibiting a slightly different pattern. The overall stability described above allowed us to perform an overall analysis over twenty years of population-based data. Finally, such an approach is applicable elsewhere since the variables used in this study are frequently collected in medical cohorts. One difficulty, however, lies in being able to benefit from specific geographical data that precisely locate the place of residence of the participants.

In addition to guiding interventions in their nature and priority, characterizing the identified areas could further our understanding of the social and environmental determinants of SSB consumption. For example, further investigations assessing whether environmental factors associated with SSB consumption (e.g., density of advertising^43^ and density of SSBs sales point^44^) differ within and outside SSB-IF clusters could provide insights into the causality of SSB overconsumption on health consequences which remains controversial^45^.

## Conclusions

Numerous programs and interventions have been conducted to mitigate obesity prevalence and SSB consumption. More progress might be achieved by implementing locally tailored interventions targeting the most vulnerable populations.

Our fine-scale geospatial approach adds to the limited knowledge on the spatial variation of weight status and SSB consumption at a local level. We detected spatial clustering of both SSB-IF and BMI among a population of adults in the state of Geneva. The identification of specific areas presenting higher SSB-IF and, for some specific areas associated with higher BMI values, enables local legislators and public health experts to develop targeted interventions and paves the way for precision public health delivery. The allocation of resources to these populations in high need of intervention could improve the efficiency of local programs and potentially diminish resistance against SSB taxation.

## Supplementary information

Supplementary material

Supplementary Figure 1

Supplementary Figure 2

Supplementary Figure 3

Supplementary Figure 4

## Data Availability

The data analyzed in the current study are not publicly available due to the sensitive nature of geolocated individual data.

## References

[1] The GBD (2017). 2015 Obesity Collaborators. Health effects of overweight and obesity in 195 countries over 25 years. N. Engl. J. Med..

[2] Ludwig DS, Peterson KE, Gortmaker SL (2001). Relation between consumption of sugar-sweetened drinks and childhood obesity: a prospective, observational analysis. Lancet.

[3] Vereecken CA, Inchley J, Subramanian SV, Hublet A, Maes L (2005). The relative influence of individual and contextual socio-economic status on consumption of fruit and soft drinks among adolescents in Europe. Eur. J. Public Health.

[4] CDC. The CDC Guide to strategies for reducing the consumption of sugar-sweetened beverages (2010).

[5] Ventura EE, Davis JN, Goran MI (2011). Sugar content of popular sweetened beverages based on objective laboratory analysis: focus on fructose content. Obesity.

[6] Market Research on the Soft Drinks Industry. http://www.euromonitor.com/soft-drinks. Cited 9 Apr 2018.

[7] Unesda. Consumption-Unesda. https://www.unesda.eu/products-ingredients/consumption/. Cited 23 May 2018.

[8] Promotion Santé Suisse Rapport 3. https://promotionsante.ch/assets/public/documents/fr/5-grundlagen/publikationen/ernaehrung-bewegung/berichte/Rapport_003_PSCH_2013-09_-_Boissons_sucrees_et_poids_corporel_chez_les_enfants_et_les_adolescents.pdf. Cited 23 May 2018.

[9] Malik VS, Pan A, Willett WC, Hu FB (2013). Sugar-sweetened beverages and weight gain in children and adults: a systematic review and meta-analysis. Am. J. Clin. Nutr..

[10] Welsh JA, Lundeen EA, Stein AD (2013). The sugar-sweetened beverage wars. Curr. Opin. Endocrinol. Diabetes Obes..

[11] Hill JO, Wyatt HR, Peters JC (2012). Energy balance and obesity. Circulation.

[12] Xi B (2015). Sugar-sweetened beverages and risk of hypertension and CVD: a dose–response meta-analysis. Br. J. Nutr..

[13] Cohen L, Curhan G, Forman J (2012). Association of sweetened beverage intake with incident hypertension. J. Gen. Intern. Med..

[14] Larsson SC, Åkesson A, Wolk A (2014). Sweetened beverage consumption is associated with increased risk of stroke in women and men. J. Nutr..

[15] Bernstein AM, de Koning L, Flint AJ, Rexrode KM, Willett WC (2012). Soda consumption and the risk of stroke in men and women. Am. J. Clin. Nutr..

[16] Imamura F (2015). Consumption of sugar sweetened beverages, artificially sweetened beverages, and fruit juice and incidence of type 2 diabetes: systematic review, meta-analysis, and estimation of population attributable fraction. BMJ.

[17] Vargas-Garcia EJ (2017). Interventions to reduce consumption of sugar-sweetened beverages or increase water intake: evidence from a systematic review and meta-analysis. Obes. Rev..

[18] Brownell KD (2009). The public health and economic benefits of taxing sugar-sweetened beverages. N. Engl. J. Med..

[19] Auchincloss AH, Gebreab SY, Mair C, Diez Roux AV (2012). A review of spatial methods in epidemiology, 2000–2010. Annu. Rev. Public Health.

[20] Park S, McGuire LC, Galuska DA (2015). Regional differences in sugar-sweetened beverage intake among US adults. J. Acad. Nutr. Diet..

[21] Kumar GS (2014). Sugar-sweetened beverage consumption among adults–18 states, 2012. MMWR Morb. Mortal. Wkly. Rep..

[22] Han E, Powell LM (2013). Consumption patterns of sugar-sweetened beverages in the United States. J. Acad. Nutr. Diet..

[23] Paelinck JHP (2000). On aggregation in spatial econometric modelling. J. Geogr. Syst..

[24] Tamura K (2017). Geospatial clustering in sugar-sweetened beverage consumption among Boston youth. Int J. Food Sci. Nutr..

[25] Guessous I, Bochud M, Theler J-M, Gaspoz J-M, Pechère-Bertschi A (2012). 1999–2009 trends in prevalence, unawareness, treatment and control of hypertension in Geneva, Switzerland.. PLoS ONE.

[26] Marques-Vidal P, Gaspoz J-M, Theler J-M, Guessous I (2017). Twenty-year trends in dietary patterns in French-speaking Switzerland: toward healthier eating. Am. J. Clin. Nutr..

[27] Bernstein M (1994). [Nutritional balance of the diet of the adult residents of Geneva]. Soz. Praventivmed..

[28] Beer-Borst S, Costanza MC, Pechère-Bertschi A, Morabia A (2009). Twelve-year trends and correlates of dietary salt intakes for the general adult population of Geneva, Switzerland. Eur. J. Clin. Nutr..

[29] Mozaffarian D (2014). Global sodium consumption and death from cardiovascular causes. N. Engl. J. Med..

[30] Micha R (2014). Global, regional, and national consumption levels of dietary fats and oils in 1990 and 2010: a systematic analysis including 266 country-specific nutrition surveys. BMJ.

[31] Statistiques cantonales-République et canton de Genève. https://www.ge.ch/statistique/. Cited 9 Apr 2018.

[32] Wei Y, Pere A, Koenker R, He X (2006). Quantile regression methods for reference growth charts. Stat. Med..

[33] Getis A, Ord JK (2010). The analysis of spatial association by use of distance statistics. Geogr. Anal..

[34] Ord JK, Getis A (1995). Local spatial autocorrelation statistics: distributional issues and an application. Geogr. Anal..

[35] Anselin L (2010). Local indicators of spatial association-LISA. Geogr. Anal..

[36] Anselin, luc. & Bera, A. K. In: A. Ullah and D.E.A. Giles (eds) Spatial Dependence in Linear Regression Models with an Introduction to Spatial Econometrics Handbook of Applied Economic Statistics. Marcel Dekker, NY, 237–289 1998.

[37] Tukey JW (1949). Comparing individual means in the analysis of variance. Source.

[38] Marques-Vidal P (2015). Dietary intake according to gender and education: a twenty-year trend in a swiss adult population. Nutrients.

[39] Bosma H, van de Mheen HD, Borsboom GJ, Mackenbach JP (2001). Neighborhood socioeconomic status and all-cause mortality. Am. J. Epidemiol..

[40] Christakis NA, Fowler JH (2007). The spread of obesity in a large social network over 32 years. N. Engl. J. Med..

[41] Moodley G, Christofides N, Norris SA, Achia T, Hofman KJ (2015). Obesogenic environments: access to and advertising of sugar-sweetened beverages in Soweto, South Africa, 2013. Prev. Chronic Dis..

[42] Guessous I (2014). A comparison of the spatial dependence of body mass index among adults and children in a Swiss general population. Nutr. Diabetes.

[43] Lesser LI, Zimmerman FJ, Cohen DA (2013). Outdoor advertising, obesity, and soda consumption: a cross-sectional study. BMC Public Health.

[44] Wiecha JL, Finkelstein D, Troped PJ, Fragala M, Peterson KE (2006). School vending machine use and fast-food restaurant use are associated with sugar-sweetened beverage intake in youth. J. Am. Diet. Assoc..

[45] Stanhope KL (2016). Sugar consumption, metabolic disease and obesity: the state of the controversy. Crit. Rev. Clin. Lab. Sci..

