# Supplementary material for: Overlapping spatial clusters of sugar-sweetened beverage intake and body mass index in Geneva state, Switzerland
Source: Nutr Diabetes. 2019 Nov 14;9:35. doi: 10.1038/s41387-019-0102-0 (PMC6856345; doi:10.1038/s41387-019-0102-0)
Supplement: Supplementary file 1 — Supplementary material [file 41387_2019_102_MOESM1_ESM.docx]

**Overlapping spatial clusters of sugar-sweetened beverage intake and body mass index in Geneva state, Switzerland**

**SUPPLEMENTARY MATERIAL**

| **Table S1. Trends of body mass index and sugar-sweetened beverage intake across three subperiods: P1 (1995-2001), P2 (2002-2008), and P3 (2009-2014).** | | | |
| --- | --- | --- | --- |
| Variable | P1 (n = 5511) | P2 (n = 4714) | P3 (n = 5357) |
| Body mass index (kg/m^2^) | 24.7 | 24.89 | 25.07 |
| Body mass index adjusted (kg/m^2^) | 24.69 | 24.9 | 25.07 |
| Sugar-sweetened beverage intake (SSB/day) | 0.2 | 0.22 | 0.24 |
| Sugar-sweetened beverage intake adjusted (SSB/day) | 0.2 | 0.22 | 0.24 |

*The division of the global dataset into three subperiods P1 (1995-2001), P2 (2002-2008), and P3 (2009-2014) highlighted a slight increase of BMI and SSB-IF overtime, the difference is only significant between P1 and P3 for both the raw and adjusted variables (using the Tukey Multiple Comparison of Means honest significance difference with a family-wise error rate equal to 0.05) (Figure S1).*

| **Table S2. Moran's I across three subperiods: P1 (1995-2001), P2 (2002-2008), and P3 (2009-2014).** | | | |
| --- | --- | --- | --- |
| Variable | P1 (n = 5511) | P2 (n = 4714) | P3 (n = 5357) |
| Body mass index (kg/m^2^) | 0.0059** | 0.0114*** | 0.0100*** |
| Body mass index adjusted (kg/m^2^) | 0.0026 | 0.0103*** | 0.0071*** |
| Sugar-sweetened beverage intake (SSB/day) | -0.0001 | 0.0002 | 0.0004 |
| Sugar-sweetened beverage intake adjusted (SSB/day) | 0.0002 | 0.0004 | -0.0005 |

*Calculation of spatial autocorrelation with spatial statistic Moran’s I for the three subperiods P1 (1995-2001), P2 (2002-2008), and P3 (2009-2014). Statistical significance assessment using a conditional randomization procedure using a sample of 999 permutations with α= 0.05. The absence of global spatial autocorrelation for both variables is stable during the three subperiods while the spatial distribution of local clusters of BMI and SSB-IF slightly varies (SSB-IF hotspot downtown during P1 only) (Figure S2A-C).*

**Supplementary Figures**

**Figure S1**: Distribution of BMI (A), adjusted BMI (B), SSB-IF (C), and adjusted SSB-IF (D) across subperiods P1 (1995-2001), P2 (2002-2008) and P3 (2009-2014).

**Figure S2**: Spatial distribution of local clusters of BMI and SSB-IF across subperiods P1 (1995-2001) (A-B), P2 (2002-2008) (C-D) and P3 (2009-2014) (E-F).

**Figure S3**: Map presenting all the classes resulting from the combination of the different spatial autocorrelation regimes as measured by the Getis-Ord Gi clustering for raw SSB-IF with raw BMI (A), and adjusted SSB-IF with adjusted BMI (B). For clarity, Figure 3A and 3B only present the overlapped hotspots for raw SSB-IF and raw BMI and adjusted SSB-IF and adjusted BMI, respectively.

**Figure S4**: Spatial distribution of the pseudo p-value for the adjusted SSB-IF (A) and adjusted BMI (B).
