# Supplementary figures and images for: Overlapping spatial clusters of sugar-sweetened beverage intake and body mass index in Geneva state, Switzerland
Source: Nutr Diabetes. 2019 Nov 14;9:35. doi: 10.1038/s41387-019-0102-0 (PMC6856345; doi:10.1038/s41387-019-0102-0)

*Figure S1A*

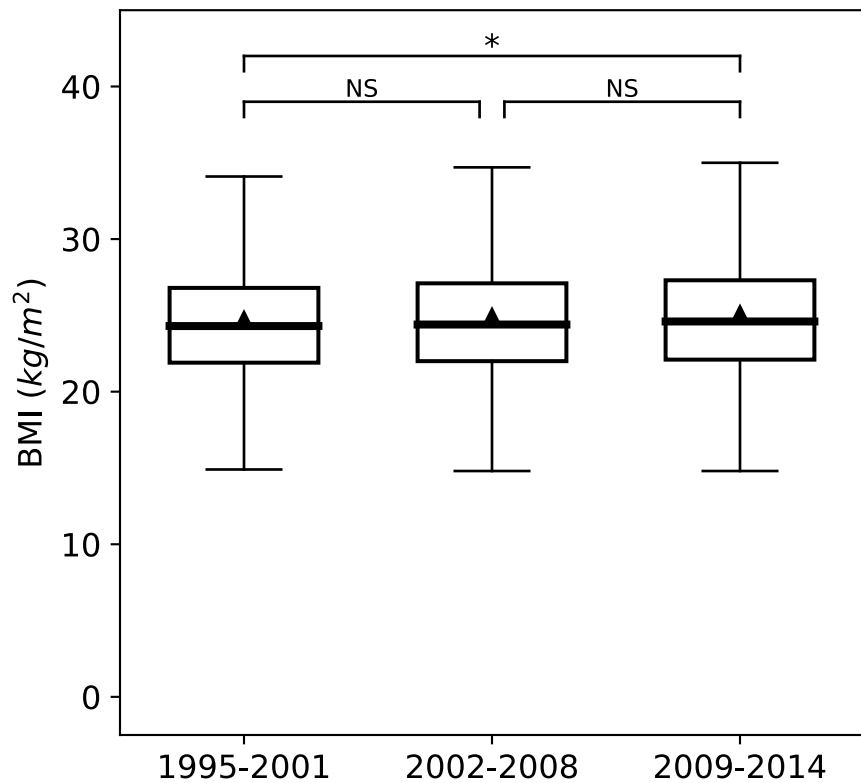

**Figure S1B**

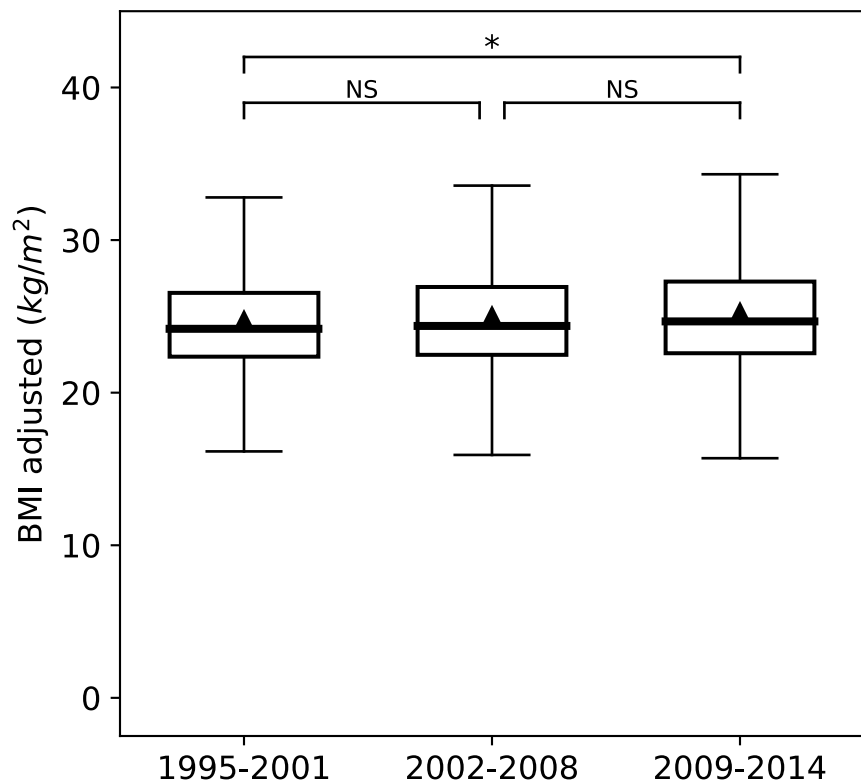

**Figure S1C**

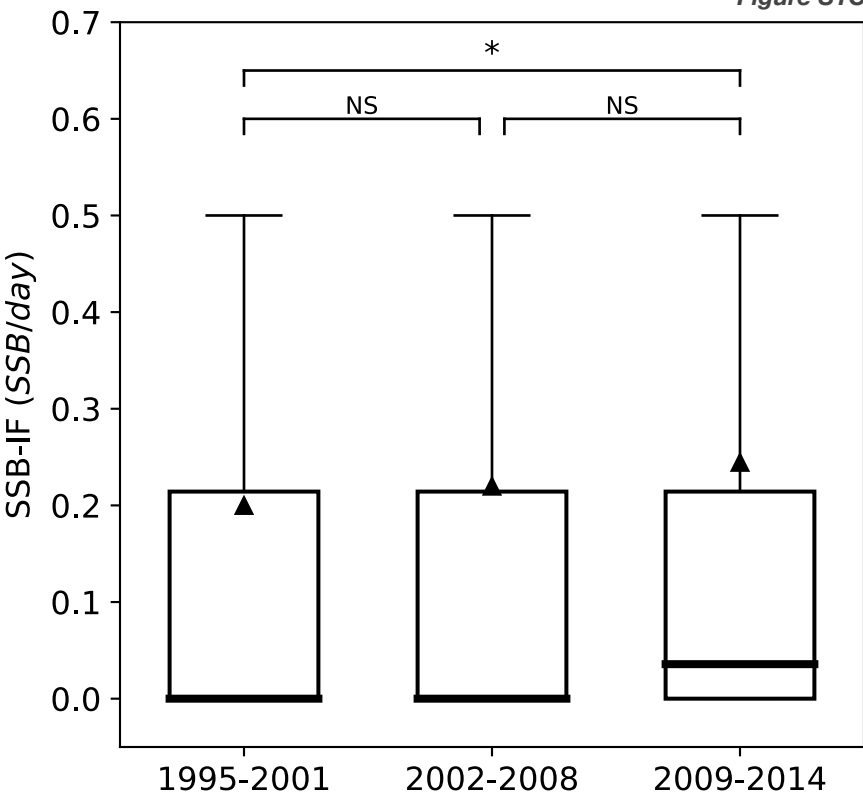

**Figure S1D**

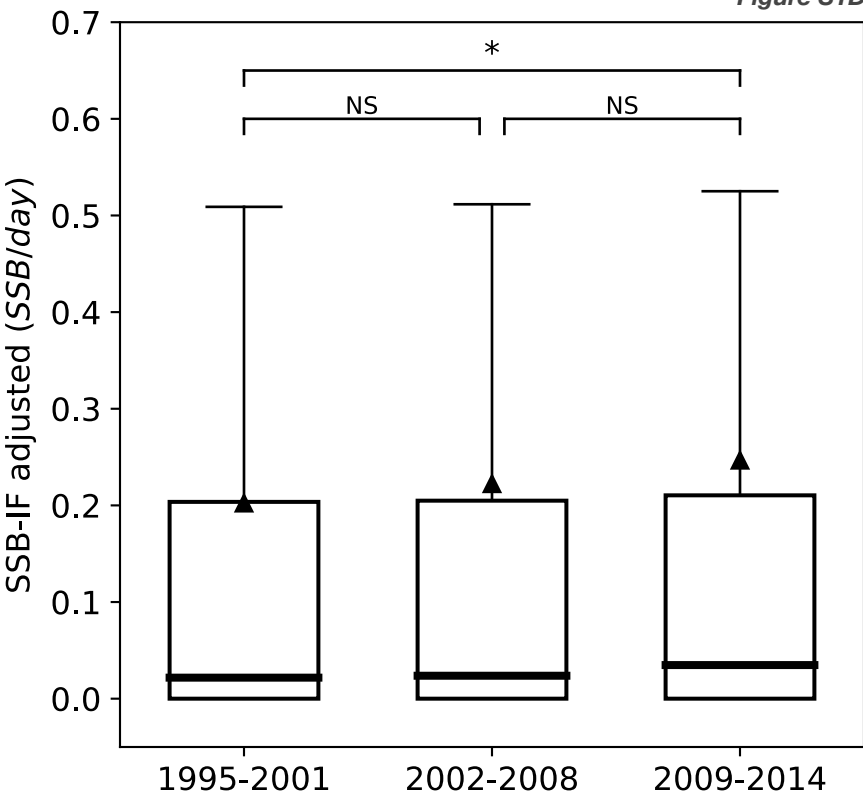

Supplement: Supplementary file 2 — Supplementary Figure 1 [file 41387_2019_102_MOESM2_ESM.pdf]
