# Supplementary material for: Overlapping spatial clusters of sugar-sweetened beverage intake and body mass index in Geneva state, Switzerland
Source: Nutr Diabetes. 2019 Nov 14;9:35. doi: 10.1038/s41387-019-0102-0 (PMC6856345; doi:10.1038/s41387-019-0102-0)
Supplement: Supplementary file 3 — Supplementary Figure 2 [file 41387_2019_102_MOESM3_ESM.pdf]

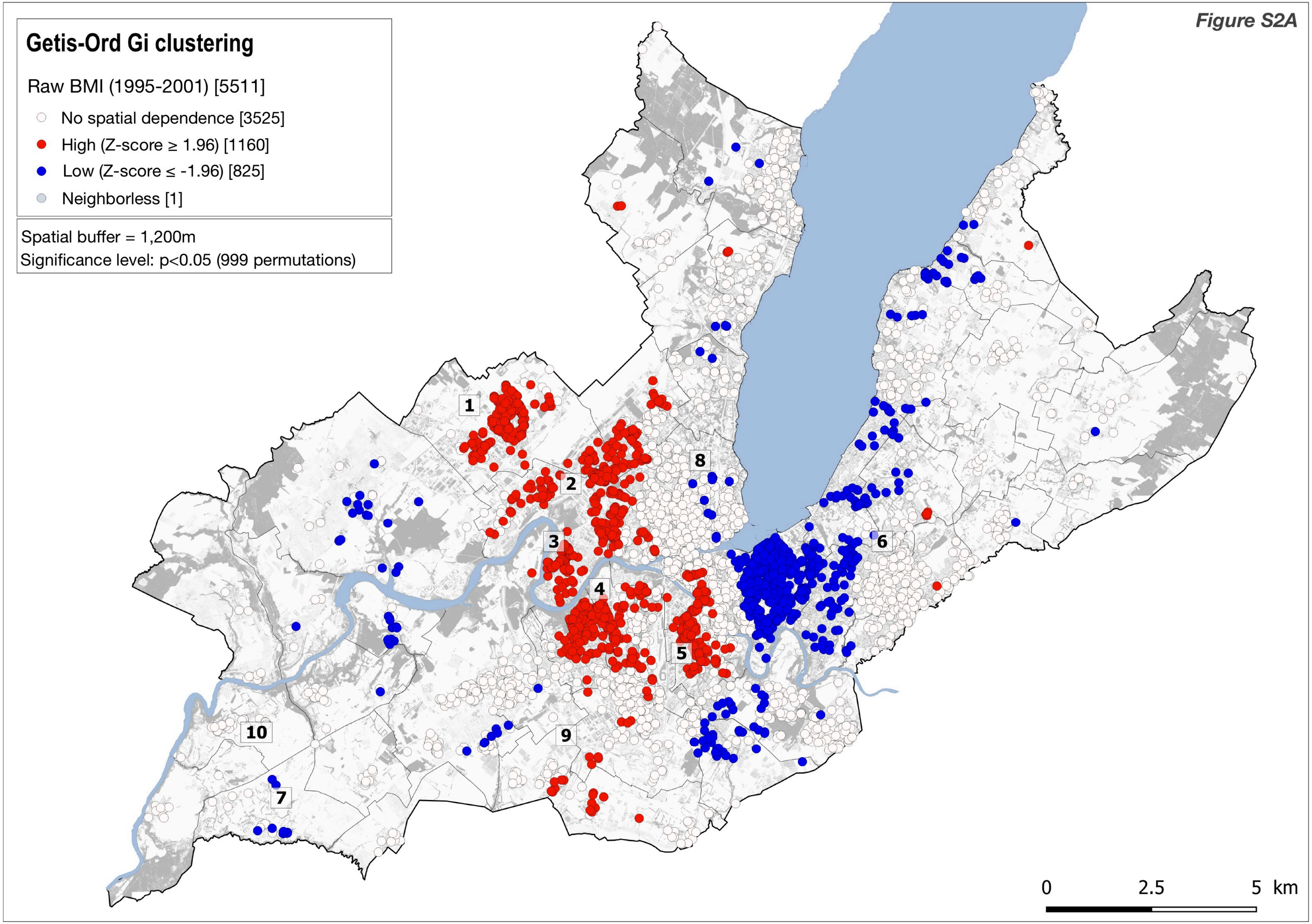
Figure S2A
Getis-Ord Gi clustering
Raw BMI (1995-2001) [5511]
No spatial dependence [3525]
High (Z-score ≥ 1.96) [1160]
Low (Z-score ≤ -1.96) [825]
Neighborless [1]
Spatial buffer = 1,200m
Significance level: p<0.05 (999 permutations)
1
2
3
4
5
6
7
8
9
10
0
2.5
5 km

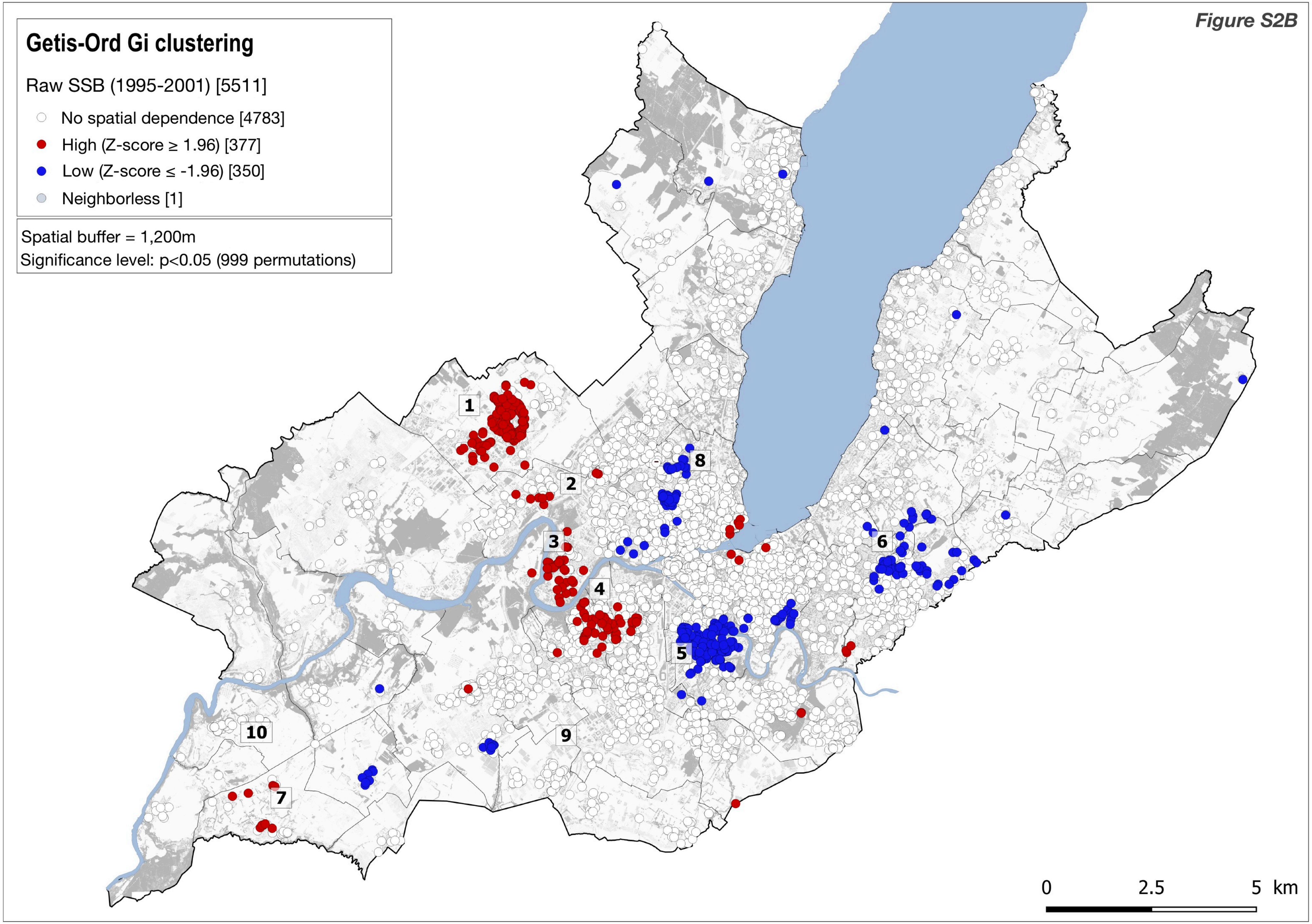
Figure S2B
Getis-Ord Gi clustering
Raw SSB (1995-2001) [5511]
No spatial dependence [4783]
High (Z-score ≥ 1.96) [377]
Low (Z-score ≤ -1.96) [350]
Neighborless [1]
Spatial buffer = 1,200m
Significance level: p<0.05 (999 permutations)
1
2
3
4
5
6
7
8
9
10
0
2.5
5 km

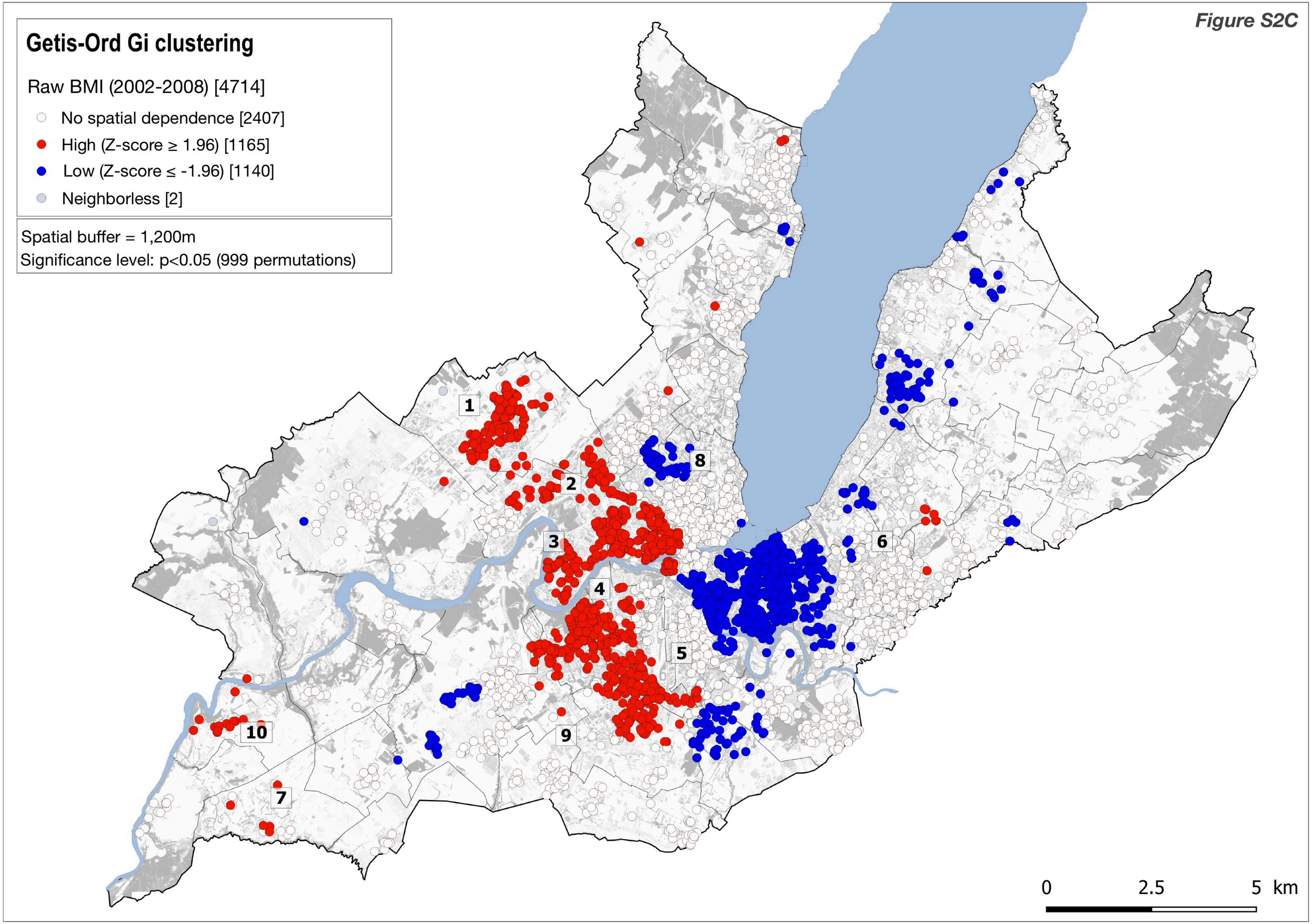

Figure S2C
Getis-Ord Gi clustering
Raw BMI (2002-2008) [4714]
No spatial dependence [2407]
High (Z-score ≥ 1.96) [1165]
Low (Z-score ≤ -1.96) [1140]
Neighborless [2]
Spatial buffer = 1,200m
Significance level: p<0.05 (999 permutations)
1
2
3
4
5
6
7
8
9
10
0
2.5
5 km

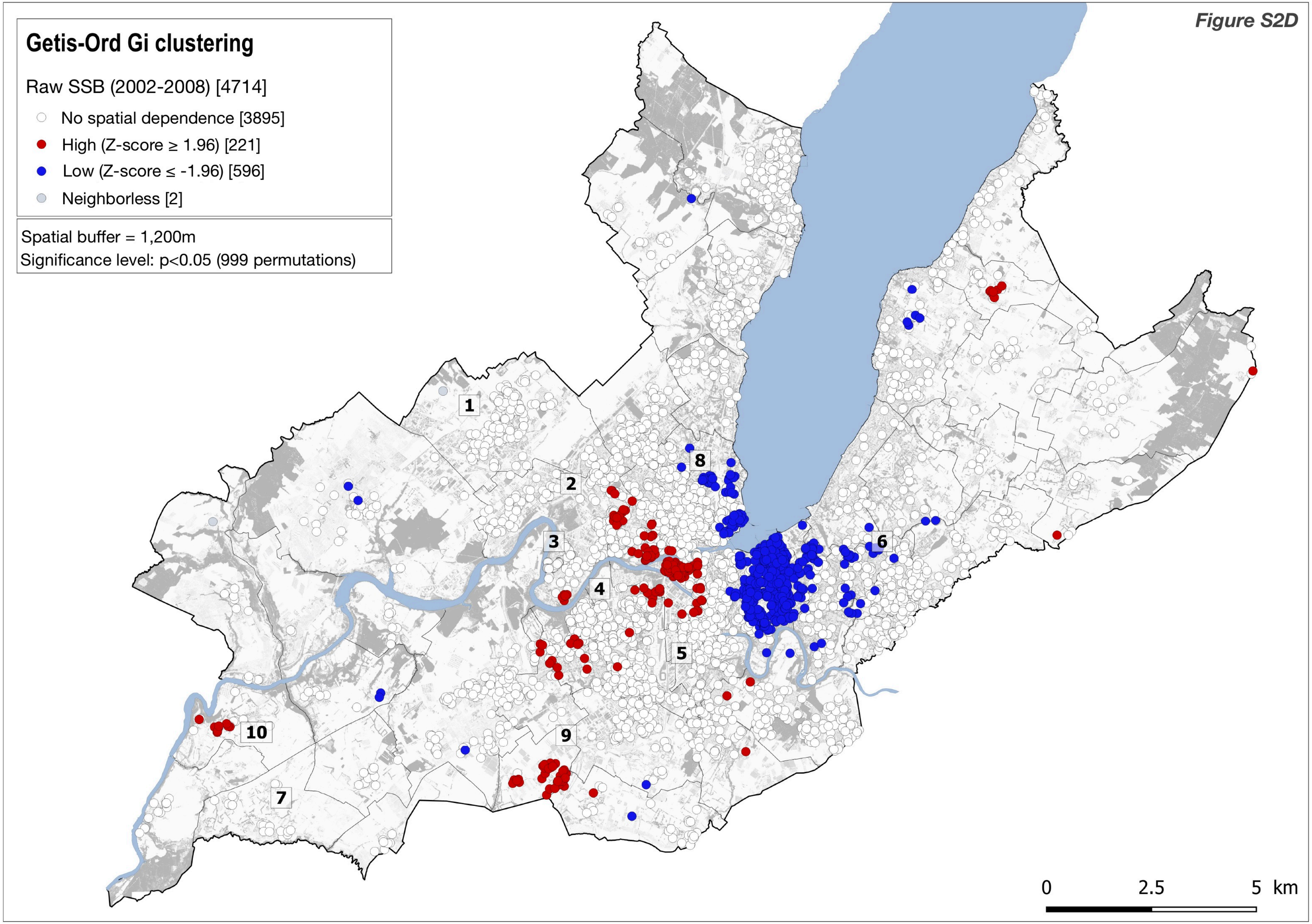
Figure S2D
Getis-Ord Gi clustering
Raw SSB (2002-2008) [4714]
No spatial dependence [3895]
High (Z-score ≥ 1.96) [221]
Low (Z-score ≤ -1.96) [596]
Neighborless [2]
Spatial buffer = 1,200m
Significance level: p<0.05 (999 permutations)
1
2
3
4
5
6
7
8
9
10
0
2.5
5 km

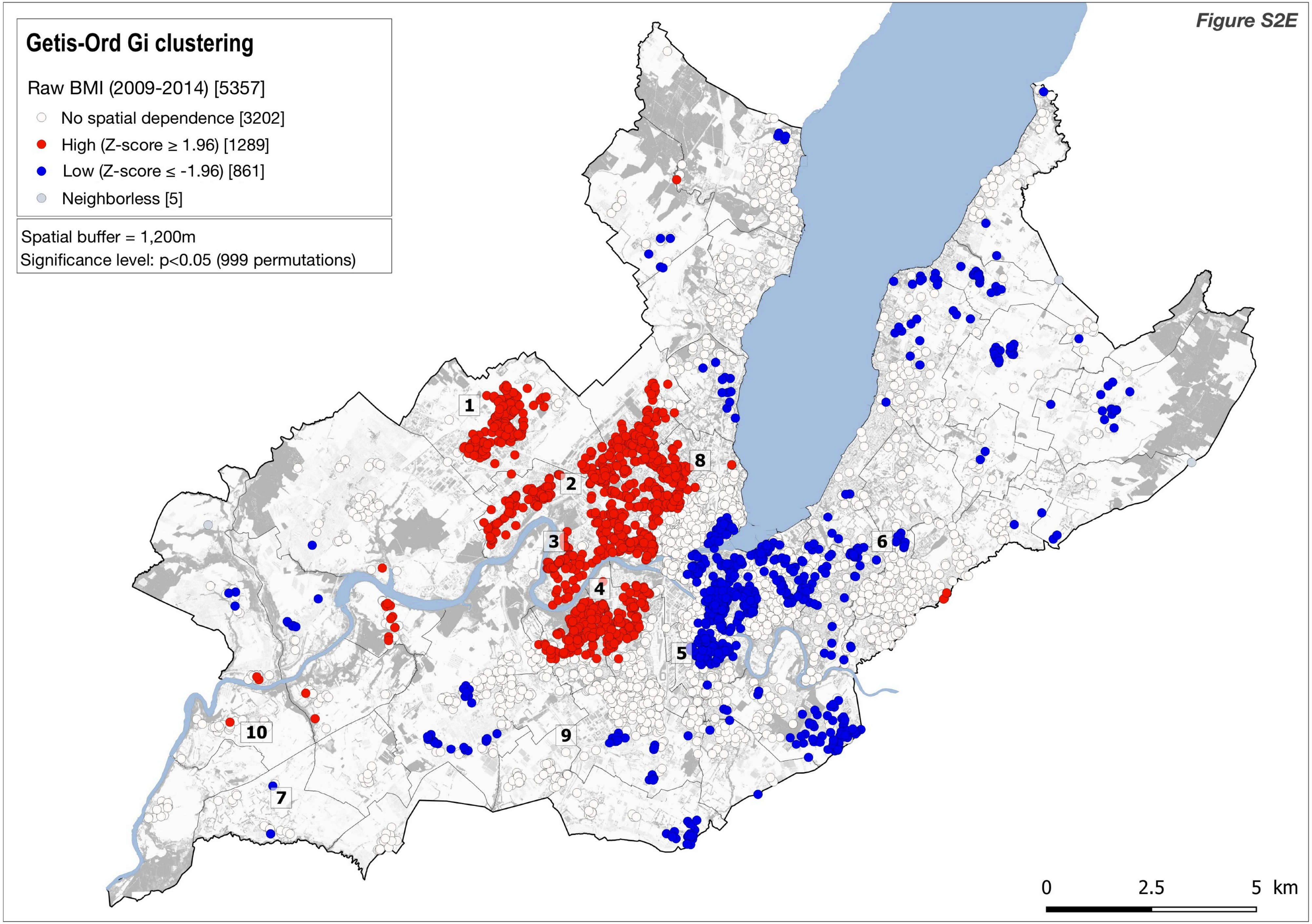
Figure S2E
Getis-Ord Gi clustering
Raw BMI (2009-2014) [5357]
No spatial dependence [3202]
High (Z-score ≥ 1.96) [1289]
Low (Z-score ≤ -1.96) [861]
Neighborless [5]
Spatial buffer = 1,200m
Significance level: p<0.05 (999 permutations)
1
2
3
4
5
6
7
8
9
10
0
2.5
5 km

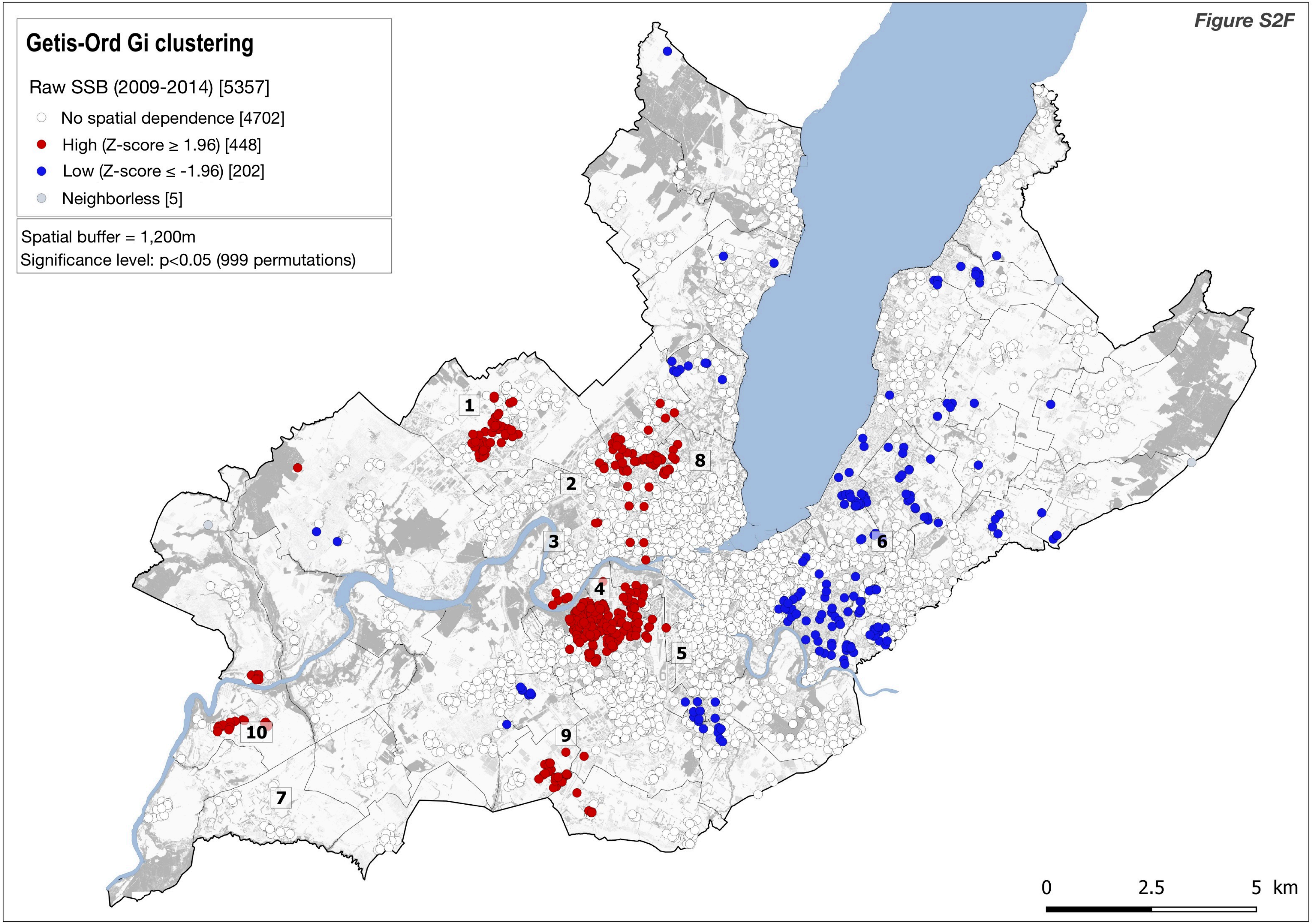
Figure S2F
Getis-Ord Gi clustering
Raw SSB (2009-2014) [5357]
No spatial dependence [4702]
High (Z-score ≥ 1.96) [448]
Low (Z-score ≤ -1.96) [202]
Neighborless [5]
Spatial buffer = 1,200m
Significance level: p<0.05 (999 permutations)
1
2
3
4
5
6
7
8
9
10
0
2.5
5 km
