# Supplementary material for: Overlapping spatial clusters of sugar-sweetened beverage intake and body mass index in Geneva state, Switzerland
Source: Nutr Diabetes. 2019 Nov 14;9:35. doi: 10.1038/s41387-019-0102-0 (PMC6856345; doi:10.1038/s41387-019-0102-0)
Supplement: Supplementary file 4 — Supplementary Figure 3 [file 41387_2019_102_MOESM4_ESM.pdf]

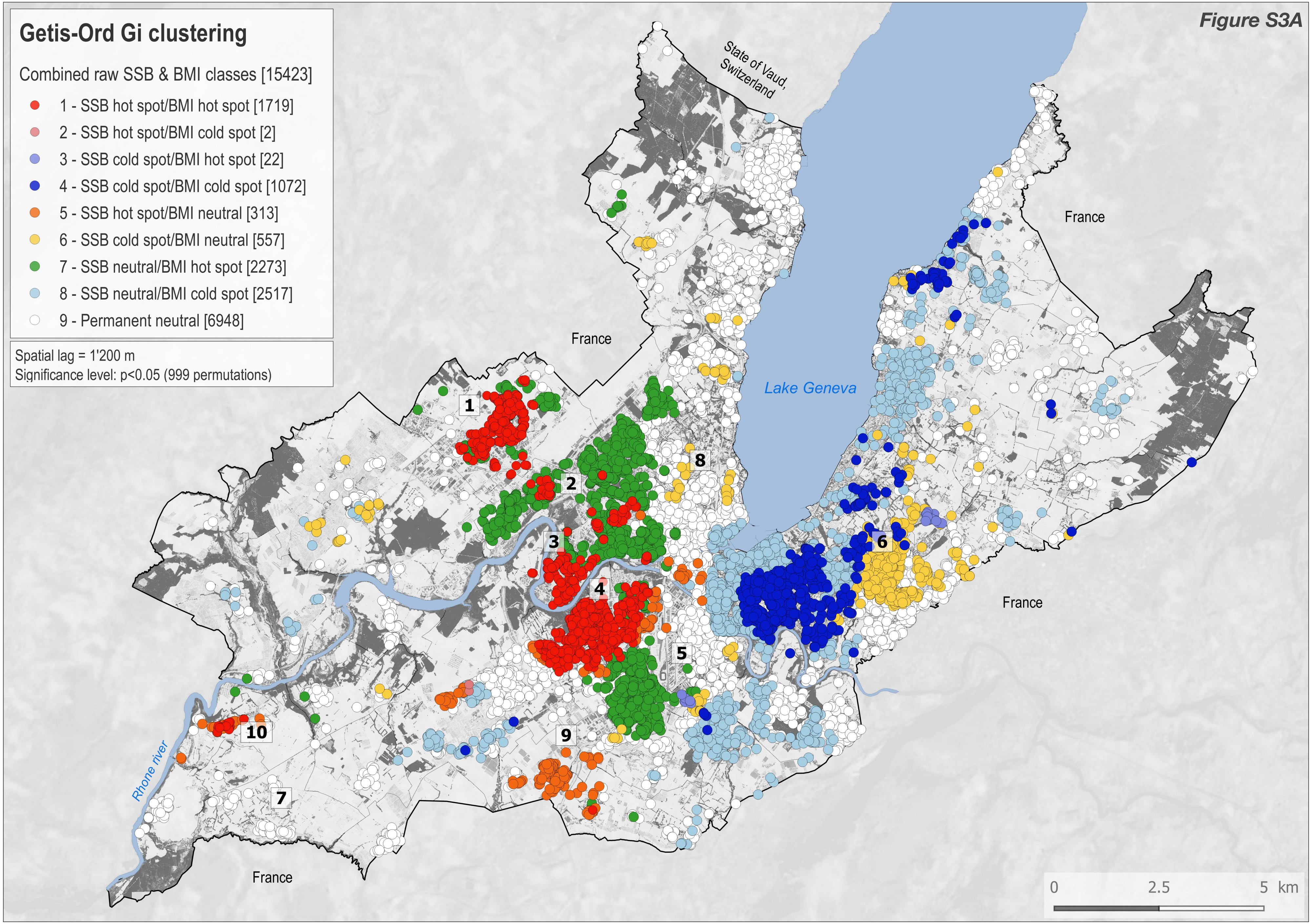

Figure S3A
Getis-Ord Gi clustering
Combined raw SSB & BMI classes [15423]
1 - SSB hot spot/BMI hot spot [1719]
2 - SSB hot spot/BMI cold spot [2]
3 - SSB cold spot/BMI hot spot [22]
4 - SSB cold spot/BMI cold spot [1072]
5 - SSB hot spot/BMI neutral [313]
6 - SSB cold spot/BMI neutral [557]
7 - SSB neutral/BMI hot spot [2273]
8 - SSB neutral/BMI cold spot [2517]
9 - Permanent neutral [6948]
Spatial lag = 1'200 m
Significance level: p<0.05 (999 permutations)
State of Vaud, Switzerland
France
Lake Geneva
France
France
France
Rhone river
France
1
2
3
4
5
6
7
8
9
10
0
2.5
5 km

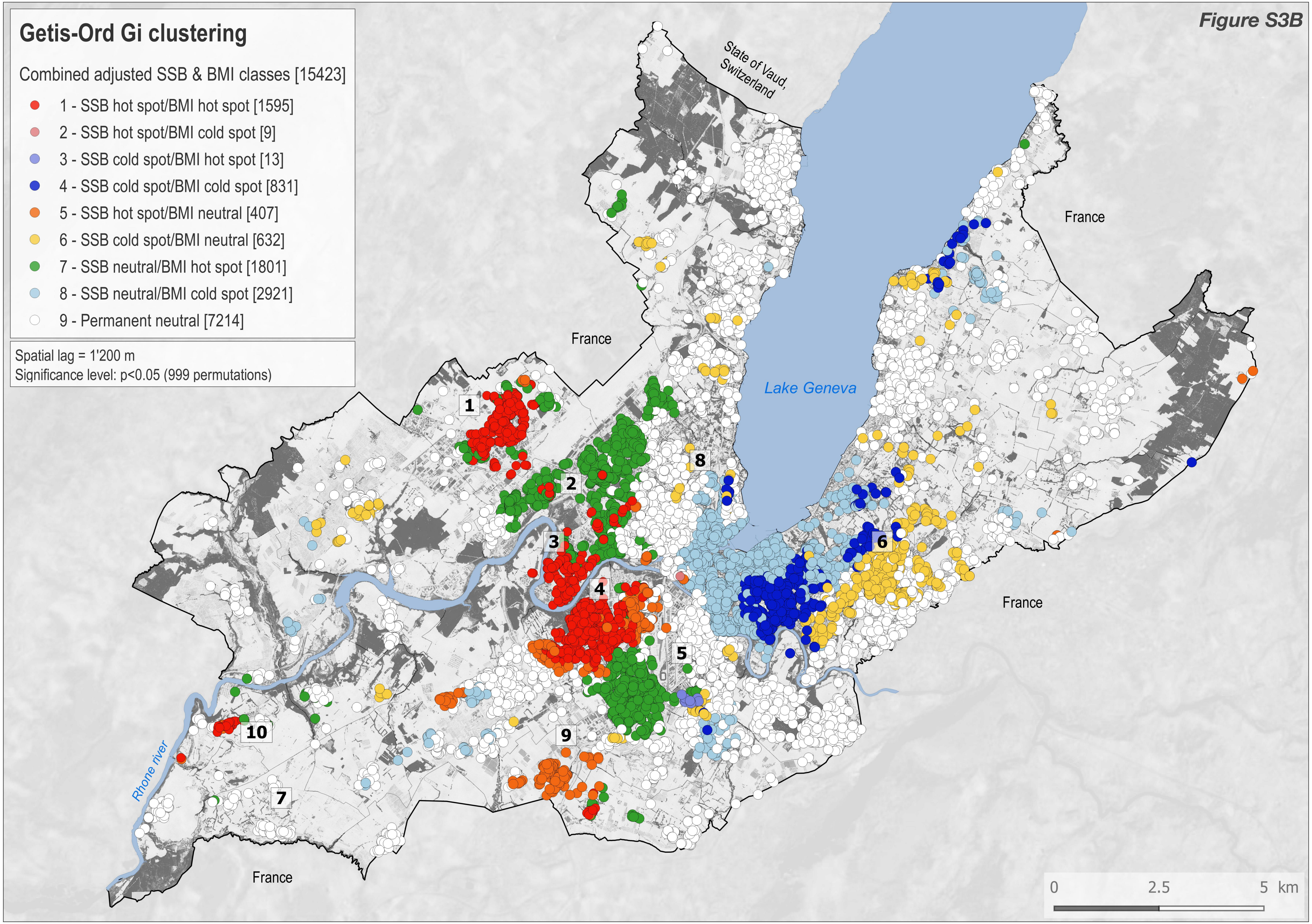

Figure S3B
Getis-Ord Gi clustering
Combined adjusted SSB & BMI classes [15423]
1 - SSB hot spot/BMI hot spot [1595]
2 - SSB hot spot/BMI cold spot [9]
3 - SSB cold spot/BMI hot spot [13]
4 - SSB cold spot/BMI cold spot [831]
5 - SSB hot spot/BMI neutral [407]
6 - SSB cold spot/BMI neutral [632]
7 - SSB neutral/BMI hot spot [1801]
8 - SSB neutral/BMI cold spot [2921]
9 - Permanent neutral [7214]
Spatial lag = 1'200 m
Significance level: p<0.05 (999 permutations)
State of Vaud, Switzerland
France
Lake Geneva
France
France
France
Rhone river
1
2
3
4
5
6
7
8
9
10
0
2.5
5 km
