# Supplementary material for: Overlapping spatial clusters of sugar-sweetened beverage intake and body mass index in Geneva state, Switzerland
Source: Nutr Diabetes. 2019 Nov 14;9:35. doi: 10.1038/s41387-019-0102-0 (PMC6856345; doi:10.1038/s41387-019-0102-0)
Supplement: Supplementary file 5 — Supplementary Figure 4 [file 41387_2019_102_MOESM5_ESM.pdf]

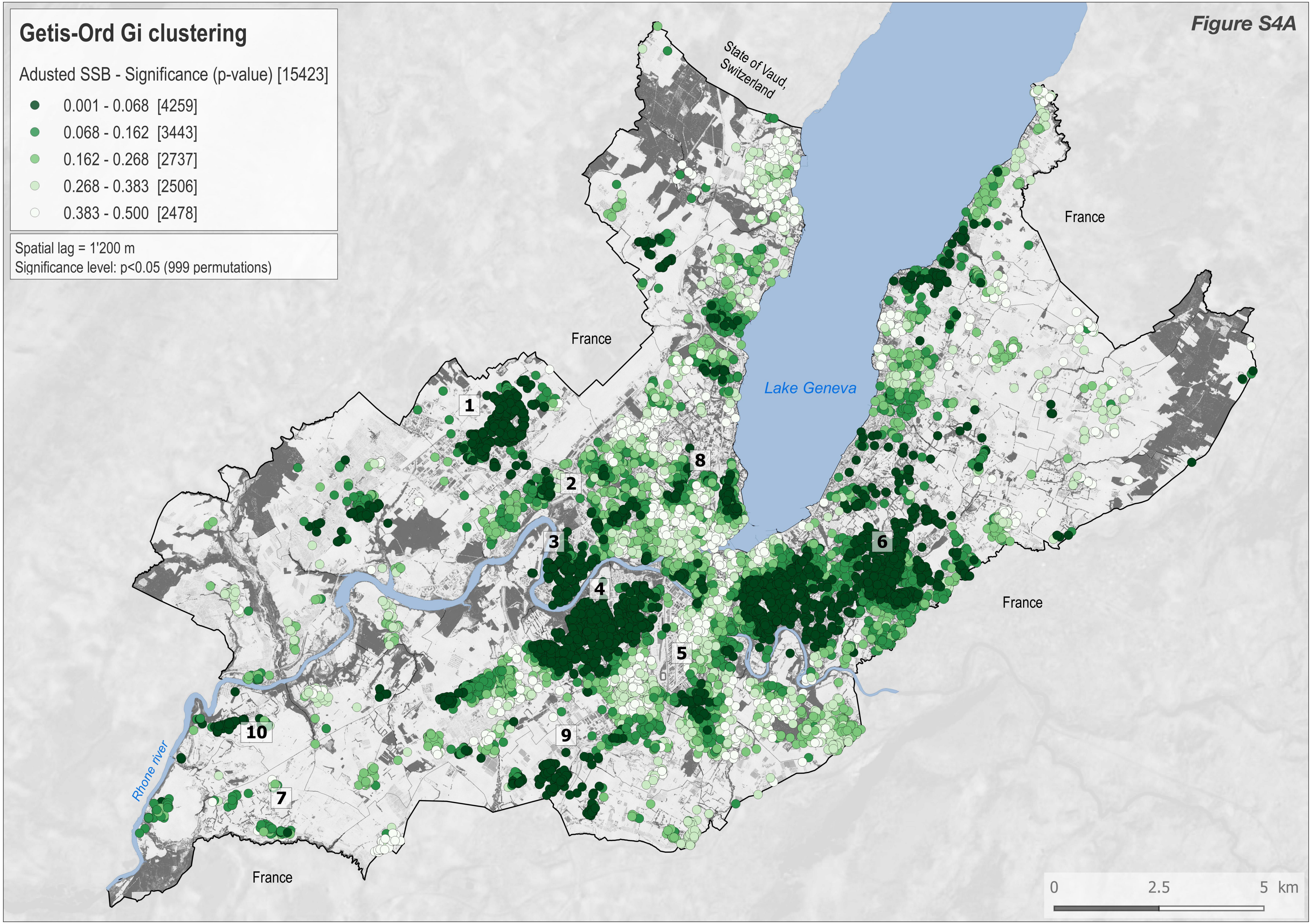
Getis-Ord Gi clustering
Adusted SSB - Significance (p-value) [15423]
0.001 - 0.068 [4259]
0.068 - 0.162 [3443]
0.162 - 0.268 [2737]
0.268 - 0.383 [2506]
0.383 - 0.500 [2478]
Spatial lag = 1'200 m
Significance level: p<0.05 (999 permutations)
Figure S4A
State of Vaud, Switzerland
France
Lake Geneva
France
France
France
Rhone river
1
2
3
4
5
6
7
8
9
10
0
2.5
5 km

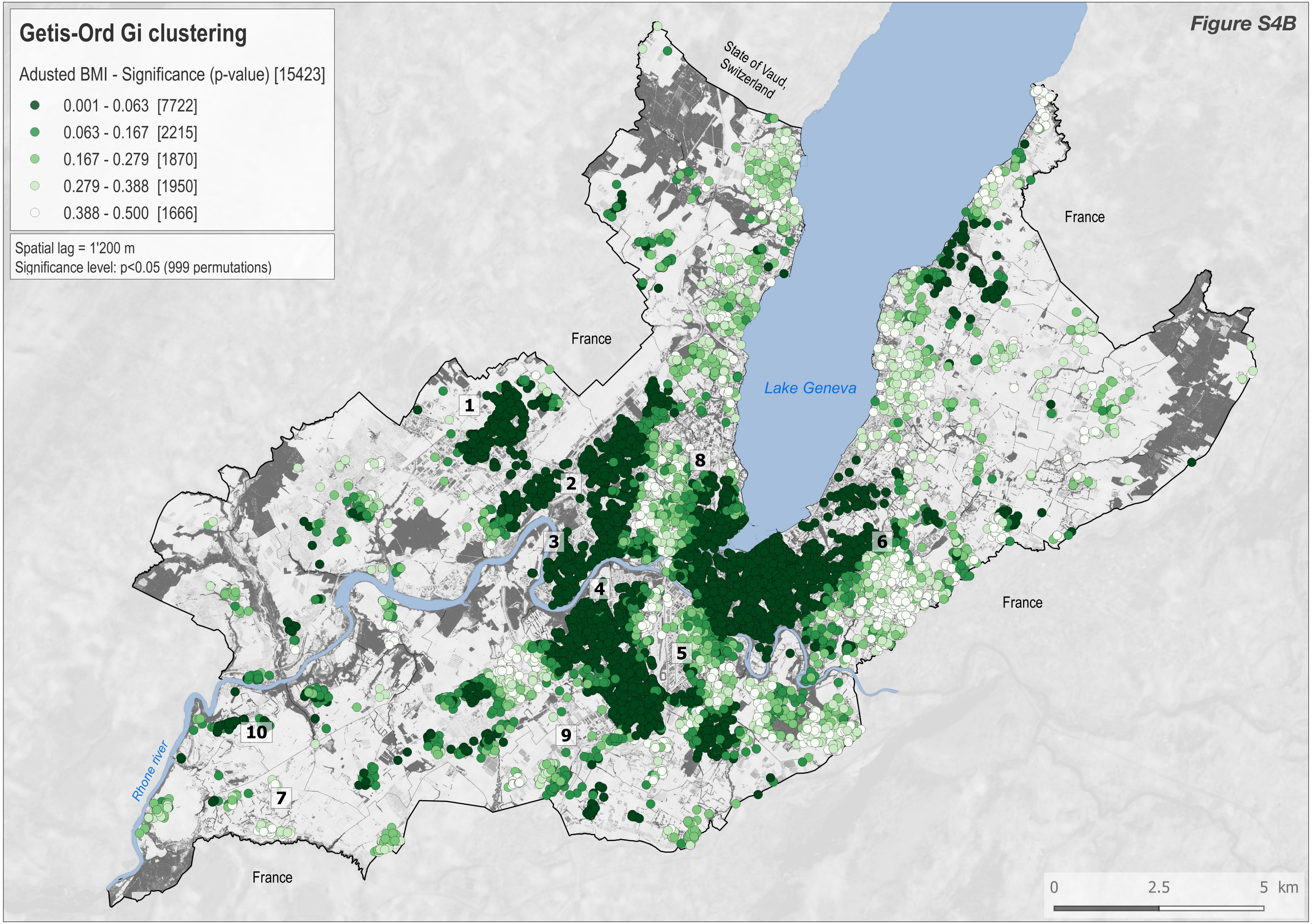

Figure S4B
Getis-Ord Gi clustering
Adusted BMI - Significance (p-value) [15423]
0.001 - 0.063 [7722]
0.063 - 0.167 [2215]
0.167 - 0.279 [1870]
0.279 - 0.388 [1950]
0.388 - 0.500 [1666]
Spatial lag = 1'200 m
Significance level: p<0.05 (999 permutations)
State of Vaud, Switzerland
France
Lake Geneva
France
France
Rhone river
France
1
2
3
4
5
6
7
8
9
10
0
2.5
5 km
